# Supplementary material for: Modeling glioblastoma heterogeneity as a dynamic network of cell states
Source: Mol Syst Biol. 2021 Sep 16;17(9):e10105. doi: 10.15252/msb.202010105 (PMC8444284; doi:10.15252/msb.202010105)
Supplement: Supplementary file 6 — Source Data for Figure 5 [file MSB-17-e10105-s004.zip › Figure5A_sourcedata/GSEA_3071/hallmarks_stateI.GseaPreranked.1623742531165/HALLMARK_SPERMATOGENESIS.html]

Details for gene set HALLMARK\_SPERMATOGENESIS[GSEA]

|  || Dataset | state13071 |
| Phenotype | NoPhenotypeAvailable |
| Upregulated in class | na\_neg |
| GeneSet | HALLMARK\_SPERMATOGENESIS |
| Enrichment Score (ES) | -0.44967526 |
| Normalized Enrichment Score (NES) | -1.751541 |
| Nominal p-value | 0.022690438 |
| FDR q-value | 0.028810536 |
| FWER p-Value | 0.243 |
Table: GSEA Results Summary

  

Fig 1: Enrichment plot: HALLMARK\_SPERMATOGENESIS      
 Profile of the Running ES Score & Positions of GeneSet Members on the Rank Ordered List

  

| PROBE | GENE SYMBOL | GENE\_TITLE | RANK IN GENE LIST | RANK METRIC SCORE | RUNNING ES | CORE ENRICHMENT || 1 | TALDO1 |  |  | 455 | 0.545 | -0.0966 | No |
| 2 | SLC12A2 |  |  | 903 | 0.350 | -0.2032 | No |
| 3 | NPHP1 |  |  | 1140 | 0.300 | -0.2523 | No |
| 4 | GSTM3 |  |  | 1182 | 0.292 | -0.2459 | No |
| 5 | ARL4A |  |  | 1195 | 0.289 | -0.2313 | No |
| 6 | TTK |  |  | 1620 | -0.270 | -0.3362 | No |
| 7 | VDAC3 |  |  | 1728 | -0.284 | -0.3492 | No |
| 8 | KIF2C |  |  | 1735 | -0.284 | -0.3332 | No |
| 9 | CCNB2 |  |  | 1757 | -0.288 | -0.3213 | No |
| 10 | AGFG1 |  |  | 1858 | -0.300 | -0.3313 | No |
| 11 | SCG3 |  |  | 2025 | -0.322 | -0.3589 | No |
| 12 | HSPA4L |  |  | 2105 | -0.335 | -0.3606 | No |
| 13 | BUB1 |  |  | 2416 | -0.396 | -0.4250 | Yes |
| 14 | STRBP |  |  | 2420 | -0.397 | -0.4010 | Yes |
| 15 | AURKA |  |  | 2467 | -0.404 | -0.3890 | Yes |
| 16 | PARP2 |  |  | 2542 | -0.425 | -0.3837 | Yes |
| 17 | DCC |  |  | 2643 | -0.451 | -0.3843 | Yes |
| 18 | JAM3 |  |  | 2654 | -0.455 | -0.3587 | Yes |
| 19 | SPATA6 |  |  | 2882 | -0.531 | -0.3908 | Yes |
| 20 | NF2 |  |  | 2972 | -0.583 | -0.3800 | Yes |
| 21 | NCAPH |  |  | 2974 | -0.585 | -0.3437 | Yes |
| 22 | TOPBP1 |  |  | 3033 | -0.621 | -0.3216 | Yes |
| 23 | PEBP1 |  |  | 3069 | -0.650 | -0.2911 | Yes |
| 24 | DBF4 |  |  | 3117 | -0.690 | -0.2615 | Yes |
| 25 | RFC4 |  |  | 3129 | -0.701 | -0.2209 | Yes |
| 26 | CDK1 |  |  | 3149 | -0.727 | -0.1809 | Yes |
| 27 | GAD1 |  |  | 3211 | -0.796 | -0.1488 | Yes |
| 28 | PIAS2 |  |  | 3240 | -0.832 | -0.1049 | Yes |
| 29 | CDKN3 |  |  | 3293 | -0.916 | -0.0626 | Yes |
| 30 | PCSK1N |  |  | 3332 | -0.981 | -0.0122 | Yes |
| 31 | EZH2 |  |  | 3342 | -1.015 | 0.0485 | Yes |
Table: GSEA details [plain text format]

  

Fig 2: HALLMARK\_SPERMATOGENESIS: Random ES distribution      
 Gene set null distribution of ES for **HALLMARK\_SPERMATOGENESIS**
